# Robot-assisted total posterior arthroplasty (TOPS)

**DOI:** 10.3171/2025.4.FOCVID2517

**Published:** 2025-07-01

**Authors:** Pegah Ghamasaee, Ataollah Shahbandi, Abdul Mounnem Yassin Kassab, Mohamad Bakhaidar, Reyna Escalante, Saman Shabani

**Affiliations:** 1 Department of Neurosurgery, Medical College of Wisconsin, Milwaukee, Wisconsin; and; 2 School of Osteopathic Medicine, University of the Incarnate Word, San Antonio, Texas

## Abstract

The authors describe the case of a patient with lumbar stenosis and neurogenic claudication who underwent a robot-assisted total posterior arthroplasty (TOPS [Total Posterior Spine] System) procedure. The TOPS is a motion-preserving implant that replaces the facet joints, allowing a wider decompression (when compared with laminectomy) and normal postoperative range of motion (as opposed to fusion), decreasing the risk of adjacent segment degeneration and the need for surgery in the future. The robot allows for precise placement of preplanned pedicle screws, allowing for accurate intraoperative device placement and decreased operative time. This patient returned to an active lifestyle without complications.

The video can be found here: https://stream.cadmore.media/r10.3171/2025.4.FOCVID2517

## Transcript

### 0:21 Introduction and Clinical Presentation.

We present the case of a robot-assisted total posterior arthroplasty. A 66-year-old female presented to the clinic with 6 months of worsening low back pain with symptoms of neurogenic claudication, characterized by throbbing pain along the posterior and lateral aspects of both legs, exacerbated by ambulation and standing. The pain was at an 8/10 at its worst. Lying down and extending her legs worsened the pain. Leaning forward or sitting down improved the pain. The patient did not have any weakness, paresthesias, or bowel or bladder symptoms. She had received an interlaminar epidural steroid injection without any relief of symptoms and had completed extensive physical therapy without improvement.

### 1:08 Physical Examination.

The patient’s motor exam showed 5/5 strength to the bilateral upper and lower extremities. Her sensation was intact to light touch throughout. She had 1+ reflexes, normal for her age, throughout the bilateral upper and lower extremities, without Hoffmann’s or clonus. She had an antalgic gait. The rest of her neurological exam was unremarkable.

### 1:30 Preoperative Imaging.

Here, we are looking at the T2-weighted sequence on the preoperative MRI of the lumbar spine showing severe canal stenosis at L4–5 with bilateral lateral recess stenosis, mild neuroforaminal stenosis, and severe facet joint effusions.

This preoperative CT was obtained with thin cuts to evaluate the bony anatomy, which better demonstrates the aforementioned facet hypertrophy at L4–5. The thin cuts of this CT allow it to be uploaded to the robot to allow for preplanning of screws and to eliminate the need for an intraoperative CT for navigation.

We then review the preoperative lumbar spine x-rays that demonstrate a stable grade 1 spondylolisthesis of L4 over L5.

### 2:22 Management Options.

The management of lumbar stenosis has both nonoperative and operative options. Nonoperative options for this patient included extensive physical therapy and epidural steroid injections, neither of which provided the patient with long-lasting symptom relief.

Surgical options include laminectomy alone, posterior lumbar fusion (with or without interbody fusion), and lumbar arthroplasty.

### 2:51 Rationale for Choosing Lumbar Arthroplasty.

Performing a laminectomy in the setting of the patient’s severe facet arthropathy and grade 1 spondylolisthesis could lead to postoperative instability, leading to worsening back pain and the possible need for fusion in the future.^1^

Performing a posterior lumbar fusion, with or without interbody fusion, would eliminate motion across the fused segments, increasing the risk of adjacent segment disease, which may necessitate further surgical intervention in the future.^1–3^ Our patient is young and active with a stable spondylolisthesis, so we did not feel that a fusion was in her best interest.

This leaves us with an arthroplasty. This would allow for us to perform a wide laminectomy and bilateral foraminotomies while avoiding fusion but still providing stability. The arthroplasty preserves range of motion, decreases the risk of development of adjacent segment disease and, thus, the need for further surgical intervention.^4,5^

### 3:57 Management Decision.

After detailed discussion with the patient, the decision was made to proceed with lumbar arthroplasty using the TOPS (Total Posterior Spine) System. We utilized robotic guidance to assist with screw planning and placement. The TOPS System is a mechanical device installed between the vertebrae after decompression. It functions as a mobile joint maintaining flexion and extension that is typically restricted by traditional spinal fusion. The device aims to reduce the risk of adjacent segment degeneration and deliver superior clinical outcomes in terms of pain relief and functional mobility compared to spinal fusion.

### 4:42 Benefits of Robot-Assisted Arthroplasty.

Using a robot allows us to preplan the screw depth on the preoperative CT scan, allowing for optimal placement of the TOPS motion implant. Following the plan intraoperatively ensures that the TOPS motion implant settles into the tulip heads without the need for further adjustment, resulting in shorter operative times. Additionally, this can help in avoiding the potential need to convert to fusion due to misalignment of the TOPS with the tulip heads.

### 5:13 Patient Positioning.

We now turn our attention to the surgery. The patient was positioned prone on a Jackson table with all pressure points padded. The Medtronic Mazor robotic guidance system was then docked to the bed.

The surgical area was prepped and draped in the usual sterile fashion. The robotic arm was draped with the proprietary drape made for this specific robot.

### 5:36 Surgery Start.

X-ray was used to plan the incision. The incision was made using a 10 blade scalpel, and Bovie cautery was used to dissect down to the spine, making sure to expose the facet joints and transverse processes bilaterally.

The robot was then secured to the patient using a percutaneous pin, and the surface was mapped by the robot. The robot was then registered to the navigation system. Two x-rays were taken with a 3D marker, allowing the robot to match the preoperative surgical plan with the intraoperative anatomy. Next, we see the plan that we made prior to beginning the case, and the steps taken to integrate the intraoperative x-ray to the preoperative CT scan. The integration is performed level by level, increasing accuracy. Alternatively, an intraoperative CT scan may be obtained and uploaded to the robot, after which the screws can be planned. We prefer to obtain intraoperative x-rays and match them to the preoperative CT scan because this allows for planning of the screws preoperatively and, with our operating room workflow, is a more time-efficient option that decreases patients’ time under anesthesia.

### 7:00 Use of Robot.

The robot arm was then moved to the location of the first planned screw trajectory where a sleeve and dilator were placed onto the entry point, shielding surrounding tissue from unnecessary trauma. A high-speed drill was used to drill a pilot hole, and the tap was used under navigated guidance to prep the pedicle. Note the white target sign that signals when the navigated tap has reached the preplanned depth. These steps were repeated until all four pedicles were prepared. The screws were not placed at this time to allow for unhindered decompression.

The robot arm was moved away from the surgical field, and a high-speed drill was used to perform the decompression to minimize manipulation of the spine and maintain the accuracy of the navigation. To maintain our workflow, we placed each pedicle screw under navigated guidance. Again, the robot’s navigation system signals when the screw has reached the preplanned depth, allowing for optimal placement of the screws. Due to the risk of navigation becoming inaccurate after the full facetectomies, we prefer to keep the robot arm out of the field so we can see and feel the prepared screw trajectories as we place the screws. The pedicle screws were then tested.

### 8:17 Placement of TOPS.

The trial alignment gauge was secured into the tulip heads to determine the size of the implant. The TOPS motion implant was attached to the TOPS inserter and filled with saline to its filling line. It was then seated into the tulip heads where it was secured in place using set caps. The set caps were final tightened, and the TOPS inserter was detached from the TOPS motion implant.

The wound was then closed in the usual fashion and the surgery was complete.

### 8:45 Postoperative Outcome.

On postoperative day 0, the patient had immediate relief of her neurogenic claudication symptoms upon standing. She remained neurologically intact on exam. At 6 weeks postsurgery, the patient had resolution of all symptoms without back pain, maintained normal range of motion with all movements, and was able to go back to her normal, very active lifestyle.

### 9:09 Robot-Assisted TOPS Placement Discussion.

There are some potential complications associated with robot-assisted TOPS placement. In patients with certain body habitus, integration of x-ray to preoperative CT could prove challenging due to image quality and could lead to inaccuracies with the registration. This would then require an intraoperative CT scan followed by planning of the screws, which would lead to increased time under anesthesia. The reference array can also be bumped during the case and move. Finally, after performing a decompression with full facetectomies, navigation can become less accurate during the screw placement step. It is imperative that the screws line up craniocaudally and mediolaterally. If they do not, the TOPS device cannot be placed and the procedure will have to be converted to a fusion due to the wide, destabilizing decompression that has been performed. With that said, the utilization of robot assistance with placement of the TOPS device has eliminated the need for intraoperative adjustments of the screws, prevented conversion to fusion, and allowed a streamlined, accurate placement of the device in our patients. We believe that the robot is a great tool and will play a positive role in the future of TOPS device placement.

## Disclosures

Dr. Shabani reported consulting for Spinal Elements, DePuy, and CarboFix outside the submitted work.

## Author Contributions

Primary surgeon: Shabani. Assistant surgeon: Ghamasaee, Yassin Kassab. Editing and drafting the video and abstract: Shahbandi, Ghamasaee, Bakhaidar. Critically revising the work: Shahbandi, Ghamasaee, Bakhaidar, Shabani. Reviewed submitted version of the work: Shahbandi, Bakhaidar, Escalante, Shabani. Approved the final version of the work on behalf of all authors: Shahbandi. Supervision: Bakhaidar. Illustration: Escalante.

## Supplemental Information

### Patient Informed Consent

The necessary patient informed consent was obtained in this study.
